# HLA inherence as a potential parameter in checkpoint inhibitor-associated autoimmune adverse event assessment

**DOI:** 10.3389/fmed.2023.1288844

**Published:** 2024-01-08

**Authors:** Sophia Gandarillas, Elizabeth Schoenberg Newland, Deborah Toppmeyer, Ryan Stephenson, Lisa Denzin, Bahar Dasgeb

**Affiliations:** ^1^Department of Dermatology, Wayne State University, Detroit, MI, United States; ^2^Department of Dermatology, Johns Hopkins School of Medicine, Baltimore, MD, United States; ^3^Department of Medical Oncology, Rutgers Cancer Institute of New Jersey, New Brunswick, NJ, United States; ^4^Department of Pediatrics, Child Health Institute of New Jersey, Rutgers Medical School, New Brunswick, NJ, United States; ^5^Department of Surgical Oncology, Rutgers Cancer Institute of New Jersey, New Brunswick, NJ, United States

**Keywords:** irAE, oncology, checkpoint inhibitors, HLA, HLA inherence

## Abstract

**Background:**

The success of immunotherapy has made it a lifesaving treatment, but not without side effects. Currently, the risk factors for developing immune-related adverse events (irAEs) in patients who receive immunotherapy are poorly understood, and there is no risk-stratifying mechanism for potentially fatal irAEs. It is postulated that oncology patients with preexisting autoimmune diseases are likely to have flares on immunotherapy. However, some patients develop *de novo* autoimmune conditions on immunotherapy without a prior history. Literature reports have postulated that human leukocyte antigen (HLA) inherence may play a role in irAEs. However, this potential remains underexplored.

**Methods:**

The oncology patients who developed autoimmune adverse events on immunotherapy for whom the continuation of treatment was prudent or lifesaving were selected. Of note, all nine patients received checkpoint inhibitors (CIs). Of the nine selected patients, only one had a prior history of an autoimmune condition. None of the nine selected patients had an active autoimmune condition at the time of CI initiation. Their HLA was typed, and the results were cross-referenced with the literature reports in PubMed and Google search with the corresponding autoimmune condition of each patient.

**Results:**

Herein, we report nine patients with irAEs for whom retrospective HLA typing revealed the inherence of multiple related HLA alleles that may correspond to the autoimmune condition that they had developed on immunotherapy. It is to be mentioned that the inherence of enriched disease-related HLA alleles was shared among patients with the same irAEs. These patients developed a range of irAEs including bullous pemphigoid, pemphigus foliaceus/vulgaris, thyroiditis, vitiligo, and hepatitis on immunotherapy. Although some combinations of disease-related HLA were well reported in otherwise idiopathic autoimmune diseases, a frequently repeated HLA allele combination in our patient population was found to be rarely seen in the general population.

**Conclusion:**

The authors suggest that an enriched inherence of disease-related HLA alleles may play a role in the genetic propensity for the development of irAEs in oncology patients, who receive immunotherapy, including CIs. Inherence of more than one or a cluster of particular autoimmune disease-related HLA alleles in patients who receive immunotherapy may unmask the corresponding autoimmune disease as the genotype inherence presents with the phenotype of the corresponding condition. It is suggested that enriched linked HLA genotypes, which are otherwise rare in the general population, may present as the corresponding phenotype of the autoimmune condition. Such clinical presentation, enhanced by immunotherapy, such as CIs, can play a role in risk stratifying patients for precision medicine and improve the outcome.

## What is already known on this topic

Immunotherapies are known to trigger immune-relatedadverse events (irAEs), though currently there is no way to predict who will and will not develop these serious reactions. Certain HLA types have been associated with autoimmune diseases. This study proposes that HLA typing may be a way to predict who and who will not develop these adverse events.

## What this study adds

This pilot study is a proof-of-concept study for the possible use of HLA biomarkers as a predictive tool for adverse events related to immunotherapy. We have found that indeed HLA types do correlate with patients' propensity for developing potentially fatal irAEs.

## How this study might affect research, practice, or policy

We hope this study is the beginning of a collective effort to study HLA biomarkers on a population-wide basis. Population-based studies may allow us to narrow down a few HLA subtypes that predispose to the most dangerous irAEs. HLA subtyping prior to starting immunotherapy may allow quicker diagnosis and treatment of any irAEs that arise.

## Background

Immunotherapy has significantly improved the prognosis of oncology patients. It saves and extends life, but not without side effects. Almost 60% of patients on immunotherapy experience immune-related adverse events (irAEs) (1). The risk factors for developing irAEs are poorly understood, although it is postulated that patients with preexisting autoimmune diseases are more likely to have flares on immunotherapy rather than developing a *de novo* autoimmune condition (2). However, some patients on immunotherapy develop an autoimmune condition without a prior history. The incidence of irAEs is increasing as the success of immunotherapy has made it one of the most frequently used and a pillar of oncologic treatment. Although studies have suggested that HLA inherence may play a role in irAEs, this area remains under investigation with the latest data showing that certain types of HLA alleles may be associated with organ or tissue-specific irAEs (3–7). Herein, we report nine patients with irAEs for whom retrospective HLA typing revealed inherence of enriched disease-related-HLA alleles, and only one of the nine patients had a prior history of a related autoimmune condition.

## Methods

Nine oncology patients who developed autoimmune adverse events on CI, for whom the continuation of treatment was lifesaving, were HLA typed with high resolution by blood test as part of the diagnosis and assessment workup for the corresponding presented autoimmune AE. Simultaneously, their blood samples were also tested for the presence of serologic autoantibodies related to the corresponding irAE. In cases of cutaneous irAE, skin biopsies were also done for histologic as well as direct immunofluorescent (DIF) evaluation. Of the nine patients, only one had a prior history of an autoimmune condition. None of the nine patients had an active autoimmune condition at the time of CI initiation. The results of their high-resolution HLA type and serologic auto-antibody were cross-referenced with the literature reports in PubMed and Google search with the corresponding autoimmune condition of each patient for diagnostic assessment. Additionally, for those who had skin biopsy, their histopathologic and DIF reports were concluded in the diagnostic assessment and evaluation process.

## Results

**Figure 1:**
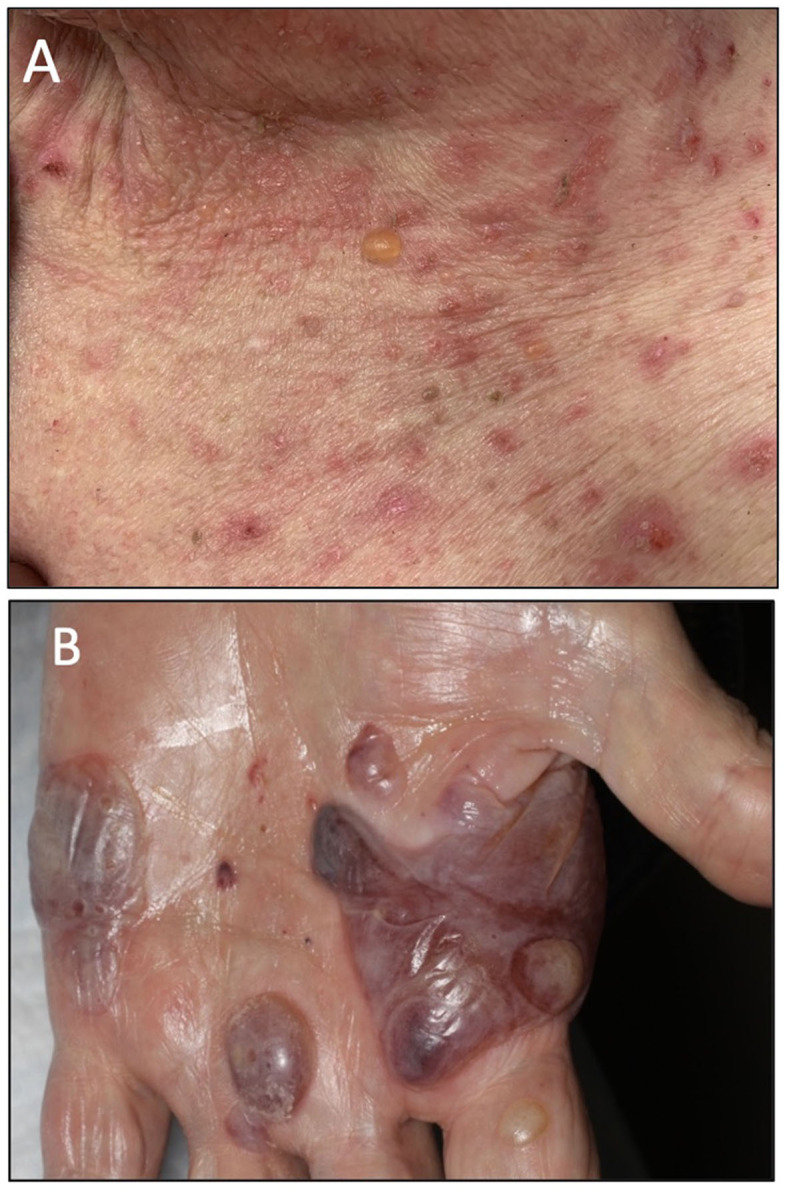
**(A)** Clinical presentation of patient A showing an intense BP blister on her chest. **(B)** Clinical presentation of patient B showing several tense hemorrhagic BP bullae on the left palm.

Patient A is a middle-aged woman with lymphatic metastatic melanoma who received monthly nivolumab for a year with a favorable response. After nine infusions, the patient developed an itchy rash with tense blisters on her upper and mid chest (Figure 1A). The patient's skin biopsy showed bullous pemphigoid (BP) and her blood workup was positive for BP-180 antibodies (Tables 1, 2). She was treated with a course of rapidly tapering prednisone, followed by daily topical triamcinolone ointment. Since the patient had a favorable response to Nivolumab and the irAE was well controlled and limited to her chest, Nivolumab was continued until there was no evidence of detectable melanoma on her restaging workup, when nivolumab was stopped. Thereafter, the BP-180 antibody became undetectable, and the blisters on her chest resolved. The high-resolution HLA typing revealed inherence of well-reported BP-associated HLA allele; DQA1 01:03 (Table 1) (8, 9). Additionally, a further enriched cluster of other BP-associated HLA alleles was also present (Table 2).

**Table 1:** The outline of patients' demographics, and their corresponding therapeutic agents, full high-resolution HLA report, and the primary tumor type.

**Patient**	**Age**	**Sex**	**Fits**	**Rx**	**Time to IrAE**	**IrAE**	**HLA**	**Tumor**
A	66	F	IV	Nivolumab	Ninth infusion	BP	HLA A 02:01 HLA A 33:01 HLA B 15:01 HLA B 53:01 HLA C 03:03 HLA C 04:01 HLA DRB3 02:02 HLA DRB1 08:04 **HLA DRB1 13**:01 **HLA DQA1 01:03** **HLA DQA1 04:01** **HLA DQB1 03**:19^*^ HLA DQB1 06:03 HLA DPA1 01:03 HLA DPA1 02:02 HLA DPB1 01:01P HLA DPB1 02:01P	Melanoma
B	75	M	III	Nivolumab	Third infusion	BP	HLA A 02:05 HLA A 03:02 HLA B 49:01 HLA B 51:01 HLA C 07:01 HLA C 15:02 **HLA DQA1 01**:04 **HLA DQA1 05:05** HLA DQB1 03:01^*^HLA DQB1 05:02 **HLA DRB1 11:04** HLA DRB1 14:01 HLA DRB3 02:01 HLA DRB3 02:24 HLA DPA1 01:03 HLA DPB1 04:01 HLA DPB1 104:01	Melanoma
C	79	M	II	Nivolumab	Fifth infusion	BP	HLA A 25:01 HLA A 32:01 HLA B 18:01 HLA B 40:02 HLA Bw 6 HLA C 02:02 HLA C 12:03 HLA DRB1 11:01 **HLA DRB1 14**:07 HLA DRB3 02:02 HLA DQB1 03:01^*^HLA DQB1 05:03 HLA DPB1 02:01 HLA DPB1 04:02	Melanoma
D	71	M	II	Ipilimumab Nivolumab	First infusion	BP	HLA A 01:01 HLA A 03:01 HLA B 07:02 **HLA B 37: 01** HLA Bw 6 HLA Bw 4 HLA C 06:02 HLA C 07:02 HLA DRB1 11:03 HLA DRB1 11:04 HLA DRB3 02:02 HLA DQB1 03^*^ HLA DPB1 15:01 HLA DPB1 104:01	Melanoma
E	88	F	II	Pembrolizumab	Third infusion	BP	HLA A 01:01 **HLA A 11:01** HLA B 35:01 **HLA B 37:01** HLA C 04:01 HLA C 06:02 HLA DRB1 01:01 HLA DRB1 11:03 HLA DRB3 02:02 **HLA DQB1 05:01** HLA DQB1 03:01^*^ HLA DPB1 04:01 HLA DPB1 04:02	Squamous cell carcinoma
F	56	F	II	Ipilimumab	Third infusion	PV	HLA A 03:01 HLA A 25:01 HLA C 03:04 HLA C 07:02 **HLA DRB1 04:01** **HLA DRB1 13:01** **HLA DQA1 01:03** **HLA DQA1 03:01** HLA DQB1 03:02^*^ HLA DQB1 06:03 HLA DPA1 02:01 HLA DPB1 01:01P HLA DPB1 17:01P	Melanoma
G	44	F	III	Pembrolizumab	First infusion	Thyroiditis	HLA-A 03:01 HLA-A 30:01 HLA B 13:02 HLA B 35:03 HLA Bw 4 HLA Bw 6 HLA C 04:01 HLA C 06: 02 **HLA DRB1 09:01** HLA DRB1 11:01 HLA DRB3 02:02 HLA DQB1 03:01^*^HLA DQB1 03:02^*^HLA DRB4 01:03 ^**^ HLA DPB1 04:02	Breast carcinoma
H	61	F	VI	Nivolumab	Fourth infusion	Thyroiditis Vitiligo	**HLA A 02:179 (T,V)** HLA A 03:01 HLA B 49:01 HLA B 53:01 **HLA Bw 4 (V)** HLA C 07:01 HLA C 07: 02 **HLA DRB1 07:01 (V)** **HLA DRB1 08:04 (T)** HLA DRB4 01:03 (T)^**^HLA DQB1 02:02 (V) HLA DQB1 03:19 (V)^*^ HLA DPB1 01:01	Melanoma
I	69	F	II	Nivolumab	First infusion	Thyroiditis Hepatitis	HLA A 02:01 HLA A 11:01 HLA B 40:01 HLA B 55:01 HLA Bw 6 HLA C 03:03 HLA C 03: 04 HLA DRB1 07:01 **HLA DRB1 04:01 (Hep)** HLA DRB3 02:02 HLA DRB4 01:03 (T)^**^ HLA DQB1 03 (Hep)^*^ HLA DPB1 04:01 HLA DPB1 10:01	Melanoma

Although a full high-resolution HLA report is provided, the HLA alleles that are found to be related to the patients' irAEs are shown in bold. For those patients who have more than one irAE, the related HLA is noted with a letter in front as follows: T, thyroiditis; V, vitiligo; Hep, hepatitis.

^*^All 9 patients with irAEs had the HLA DQB1 03 allele, reported with an enriched presence in patients with BP and other autoimmune diseases.

^**^All patients with thyroiditis had DRB4 01:03 allele.

Seven of nine patients had combined HLA DRB3 02 and DPB1alleles (highlighted in red) which are well reported in various idiopathic autoimmune diseases. All seven patients with HLA DRB3 02 and DPB1alleles showed concurrent inherence HLA DRB allele, which is a rare and uncommon finding in the general population.

**Table 2:** The outline shows the patients' serologic test results, as well as the reports of the skin histopathology and DIF if relevant.

**Patient**	**irAE**	**Histology**	**DIF**	**Serology and antibody**
A	BP	**Eosinophilic subepidermal blister**	**4+** **strong linear patterns at basement membrane C3: Positive** IgG: Negative IgM: Negative IgA: Negative	**BP180: Positive** BP230: Negative Desmoglein-1:Negative Desmoglein- 3: Negative D/C Nivolumab: BP 180: Negative
B	BP	**Eosinophilic subepidermal blister**	**Strong linear pattern at basement membrane C3: Positive IgG: Positive** IgM: negative	**BP180: Positive** BP230: Negative Desmoglein-1: Negative Desmoglein- 3: Negative Nivolumab was continued due to: limited disease lack of alternative treatment
C	BP	**Eosinophilic subepidermal blister**	Not done	**BP180: Positive** BP230: Negative Desmoglein-1:Negative Desmoglein- 3: Negative Adjuvant Nivolumab was continued due to: -mildly symptomatic clinical disease -life-extending advantage of adjuvant therapy
D	BP	**Eosinophilic subepidermal blister**	Not done	**BP180: Positive** BP230: Negative Desmoglein-1:Negative Desmoglein- 3: Negative D/C Ipi/Nivo BP180: Negative Switched to targeted therapy. The disease progressed, and Nivolumab re-started. Mild BP recurred **BP180: Positive**
E	BP	**Eosinophilic subepidermal blister**	Not done	**BP180: Positive** BP230: Negative Desmoglein-1:Negative Desmoglein- 3: Negative
F	PV	**Intraepidermal blister and acantholysis**.	**Intercellular pattern G C3: Positive IgG: Positive**	**Desmoglein-1: Positive Desmoglein-3: Intermediate** BP180: Negative BP 230: Negative D/C Ipilimumab: Desmoglein-1: Negative Desmoglein-3: Negative
G	Thyroiditis	No biopsy	Not done	Not done
H	Thyroiditis Vitiligo	No biopsy	Not done	Not done
I	Thyroiditis Hepatitis	No biopsy	Not done	Not done

Bolded items are items of particular note that demonstrate the diagnosis.

**Figure 2:**
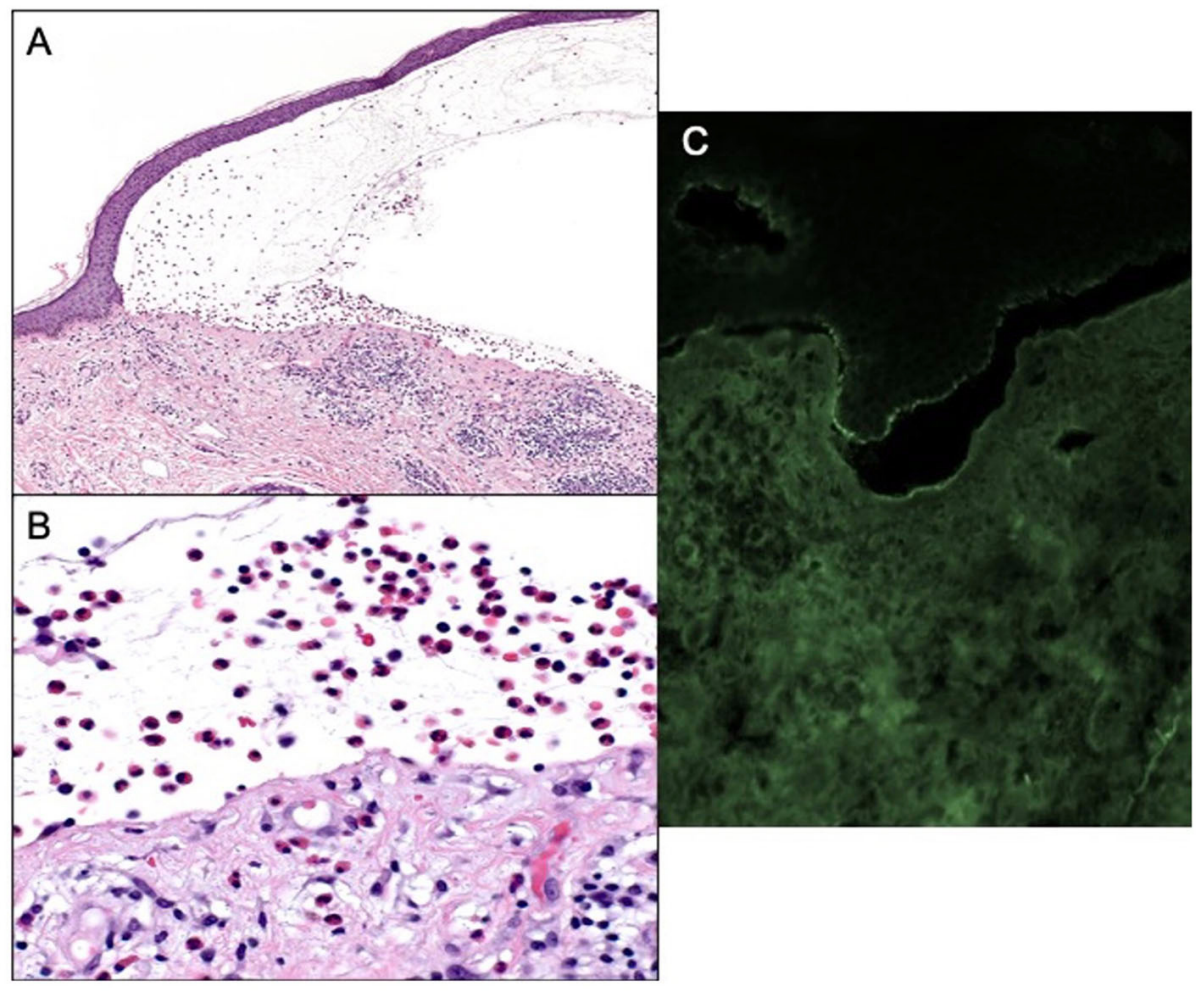
**(A)** Subepidermal blistering with an abundance of eosinophils 100× magnification and hematoxylin and eosin. **(B)** Eosinophils within blister cavity, 400× magnification. **(C)** Positive DIF, linear staining for IgG and C3 along the epidermal-dermal basement membrane.

Patient B is an elderly patient with distant metastatic melanoma on nivolumab. After the third infusion, the patient developed painful, pruritic, blood-filled bullae confined to the patient's palms and feet (Figure 1B). The fourth infusion was withheld, a skin biopsy and serologic antibodies showed BP, and the patient was treated with clobetasol under occlusion given the confined distribution of the disease involving only palms and soles (Figures 2A–C). Given patient B's significant metastatic tumor burden and prior failure of other treatment options, nivolumab was continued as a lifesaving measure in light of localized non-progressing BP. Patient B eventually passed away after a year due to tumor burden. HLA typing revealed DRB1 11:04, DQA1 05:05, and DQB1^*^03:01 (Table 1) all of which are well reported to be associated with BP (10). Once again, an enriched cluster of other BP-associated HLA alleles was also present.

Patients C and D with a diagnosis of lymphatic and distant metastatic melanoma, respectively, developed BP on immunotherapy. Patient C presented with mild urticarial BP involving < 30% body surface area which was well controlled with topical Triamcinolone. Nivolumab continued until restaging showed no detectable disease, at which point BP resolved once the immunotherapy stopped (Tables 1, 2). Patient D with distant metastatic melanoma was on combination Ipilimumab/Nivolumab (Ipi/Nivo) therapy and developed a blistering BP involving more than 30% of body surface area after the first infusion. The Ipi/Nivo was discontinued in favor of systemic targeted therapy based on the patient's tumor mutation, and the blisters resolved. Both patients C and D had skin biopsy and serology autoantibody levels consistent with the diagnosis of BP. Additionally, both patients had more than one inherent HLA allele reported to be associated with BP. It is worth mentioning that of all four aforementioned patients with pemphigoid irAE, patient D had the least number of enriched clusters of inherent BP-associated HLA alleles of only two, and just one was strongly associated with the disease, HLA DQB1^*^03:01 (10). This information became useful when patient D was considered for restarting immunotherapy when systemic targeted therapy failed and mono-immunotherapy with Nivolumab was cautiously reintroduced. New small blisters re-appeared on his chest but remained limited, and itching was well tolerated with topical Clobetasol. Of note is that all four BP patients exhibited HLA DQB1^*^03.

Patient E is an elderly patient with non-operable advanced ulcerating squamous cell carcinomas involving bilateral lower legs on pembrolizumab. After the three infusions, a pruritic bullous eruption appeared on her trunk and extremities and got worse after every infusion. A skin biopsy confirmed bullous pemphigoid and was consistent with positive serology autoantibody results. Pembrolizumab was stopped, the blisters resolved, and the patient was switched to cetuximab with favorable response. HLA typing was done, which revealed HLA A 11:01, B 37:01, DQB1 05:01, and DQB ^*^03 all reported in association with BP (8) (Table 1).

Patient F is a middle-aged adult in remission from stage III melanoma on adjuvant ipilimumab, who presented with pruritic chest lesions, which worsened with each infusion. The patient's serology and skin biopsy results (Table 2) showed pemphigus vulgaris. The lesions resolved with discontinuation of adjuvant ipilimumab followed by a decline of desmoglein antibodies to an undetectable level (Table 2). HLA typing revealed DQB1^*^0302, DQA1 0301, and DRB1 04 (Table 1), all of which have been reported in association with pemphigus (11).

Patient G is a middle-aged patient with a history of breast ductal adenocarcinoma, who developed a thyroid storm requiring hospitalization 10 days after the first pembrolizumab infusion. Patient D's TSH level dropped to 0.02 and free T4 rose to 7.72 from a normal baseline. After the pembrolizumab was discontinued, the patient's TSH and T4 eventually normalized to 3.57 and 1.67, respectively. Patient D was not investigated for thyroid autoimmunity at the time of thyroiditis. Thyroid autoantibodies (anti-TPO, anti-TSH receptor, and anti-thyroglobulin) were investigated 3.5 years later, rendering negative results (Table 1). However, HLA typing was done to risk stratify the patient for other potentially life-threatening irAEs in order to prepare for re-challenging the patient with immunotherapy due to disease recurrence. The high-resolution HLA revealed inherence of DRB 109:01, DQB1^*^03:01, DQB1^*^03:02, and DRB4, all reported to be associated with thyroid autoimmunity and diabetes (12–14). Notably, the patient has a strong family history of type I diabetes and became pre-diabetic during the treatment course.

Patient H with metastatic acral melanoma was started on nivolumab and thyroiditis presented after the patient's fourth infusion. Serology showed a TSH of 126, a free T4 of 0.3, and a T3 of 36. Nivolumab was discontinued and the patient's laboratory values improved to a TSH of 15.2 and a free T4 of 1.7. The patient's thyroid autoantibodies were tested positive at the time of thyroiditis with a TPO antibody of >900 and a thyroglobulin antibody of 1:20. HLA typing revealed DRB1 08:04, DRB4, and A 02:179 alleles, all of which have been linked to autoimmune thyroid disease (AITD) (15, 16). The patient also developed vitiligo at the same time; the high-resolution HLA typing also revealed vitiligo-associated alleles, such as HLA Bw 4, DRB1 07:01, and HLA A 02:179 among others (Table 1) (17–21).

Patient I with stage IIIB melanoma of the nose was on adjuvant nivolumab. After the first nivolumab infusion, the patient showed serologic thyroid abnormalities with a low TSH (0.006), elevated free T4 (3.09), and elevated free T3 (6.6) while remaining clinically asymptomatic. After cycle 9, thyroid serology became more abnormal with elevated TSH (16.4), low free T3 (1.4), and low free T4 (0.38) in addition to elevated ALT (166 IU/L), AST (94 IU/L), and alkaline phosphatase (291 IU/L) accompanied with abdominal pain, vomiting, and mild diarrhea, which resulted in discontinuation of Nivolumab followed by tapering of oral prednisone. Hepatitis resolved, and thyroid hormone therapy was initiated. Restating PET/CT showed no evidence of detectable melanoma. Similar to patients F and G, patient H also showed HLA DRB4, which is a well-reported allele linked with autoimmune thyroiditis. This patient also had HLA type DRB1 04:01, which is reported in association with autoimmune hepatitis (Table 1) (22, 23).

## Discussion

Checkpoint inhibitors enhance CD8 T-cell cytotoxic function by downregulating the inhibitory brakes, which can cause irAEs (24). The mechanism of irAEs is complex and occasionally life-threatening. These adverse events can occur in any organ; most commonly the skin (46–62%) and colon (22–48%) (6, 25). It is prudent to better understand such irAEs, and when possible, risk stratify patients accordingly to achieve a more precise decision at the point of care when continuation of immunotherapy is considered lifesaving (26). Though there is no current standard to predict toxicities, HLA typing has been proposed as a potential risk-stratifying parameter for irAE (26, 27). We propose that the presented patients who developed bullous pemphigoid, pemphigus vulgaris, thyroiditis, vitiligo, and hepatitis may be associated with their genetic propensity toward such autoimmune conditions, which became unmasked by immunotherapy (Table 1).

Bullous pemphigoid has been reported with almost all immunotherapies (1, 27). There are HLA associations with BP among certain populations including DQB1^*^03:01 in Caucasians and Iranians, and DQB1^*^03:02, DRB1^*^11:01, and DRB1 04:03 in the Japanese (12). In Brazilian populations, DQB1^*^ 03:01, DQA1 01:03, and DQA1 05:05 alleles have been associated with BP (9). In the northern Chinese, HLA-DQA1 05:05, DQB1 05:01, and DRB1 11:04 were found in association with BP (10). In the Han Chinese population HLA-A 11:01 and HLA-B 37:01 and in the Iranian population DQB1 05:01 have been shown to be associated with BP (8, 28). All five patients who presented with immunotherapy-associated BP had an enriched cluster of multiple HLA alleles, which at times formed a haplotype linked with BP in various population study reports. Of these alleles, the HLA DQB1^*^03 is widely reported in association with BP, in addition to other autoimmune conditions, such as alopecia areata, thyroiditis, celiac disease, colitis, and type 2 autoimmune hepatitis (28–34). It is of note that all nine patients presented here with irAEs exhibited HLA DQB1 ^*^03 allele in their HLA inherence. This is in light of the HLA DQB1^*^03 frequency of < 20% in the US with the HLA DQB1^*^03:01 frequency of 17.7% specifically. The enriched presence of alleles, such as HLA DQB1 ^*^03:01 in presented patients, in light of the low prevalence in the general population, suggests that HLA typing had the potential to be considered a biomarker to stratify irAEs in high-risk patients (35, 36).

A clinically meaningful HLA association with the development of melanoma is well reported in the literature. Additionally, some of these melanoma-linked HLA alleles are reported to overlap with those associated with immunotherapy-triggered irAEs in oncology patients. Of such linked HLAs, overlap between melanoma and CI-associated BP adverse events only includes HLA-DPB1* 01 and HLADPB1*10 which were found in patients A and I who had CI-associated BP adverse events and melanoma. Of note, neither of these alleles have been found to be associated with poor outcomes in melanoma (37). It may warrant further investigation and larger data to determine if melanoma patients with the aforementioned HLA may have a clinically meaningful risk of developing BP adverse events on immunotherapy, including CI.

Notably, all nine presented patients had HLA DQB1^*^03 allele. This allele is not only well reported with an enriched presence in BP patients, but also has been found to be linked to many other autoimmune conditions, including alopecia areata, celiac disease, and type 2 autoimmune hepatitis (29–34). Additionally, the HLA DRB4 01:03 allele, well known to be linked with autoimmune thyroiditis, was present in all 3 thyroiditis patients presented here.

Immunotherapy-associated PV is well reported in the literature with recent studies reporting the first case of PV triggered by ipilimumab (11, 25, 38). The HLA typing of patient F revealed multiple PV-associated HLA alleles, including but not limited to the DQB1^*^03:02 and DQA1 03:01 (12–14, 39–41). Such an enriched inheritance of a multiple disease-associated genotype lead to the suggestion that the enhancement of the immune system by immunotherapy may unmask an otherwise underlying dormant disease to clinical presentation via HLA (42–44).

Thyroid toxicity has also been well reported with immunotherapy (45). The incidence of thyroid irAE due to pembrolizumab is reported in ~17% and usually presents within a few weeks after the first dose. Progression from thyrotoxicosis to hypothyroidism post-immunotherapy is almost universal (46).

Patients G and H had multiple thyroiditis-associated HLA alleles including DRB1 09:01, reported in the Japanese with Hashimoto's thyroiditis, DQB1^*^03:01 reported in the Caucasians, and DQB1^*^03:02 in the Greek population with autoimmune thyroiditis (13, 14). Interestingly, DQB1^*^03:01 and ^*^03:02, detected in patient G, are known to present a shared genetic predisposition for type 1 diabetes and autoimmune thyroid disease (41), shedding light in part on the strong family history of diabetes in patient G (41, 47). Patient H possessed multiple predisposing autoimmune thyroiditis HLA alleles including HLA A2 and DRB1 08:04, which have been linked with Hashimoto's thyroiditis and Graves' disease, with the latter being reported with early onset of the disease. The HLA DRB4 seen in patient I is also well reported with Hashimoto's thyroiditis and other autoimmune thyroid conditions (48). In oncology patients, it is reported that time to thyrotoxicosis occurs within 6 weeks of the first immunotherapy infusion (15, 16).

Vitiligo is also well reported as an irAE of immunotherapy, especially in melanoma patients treated with PD-1 inhibitors with an incidence ranging from 7.5 to 11% (1). The mechanism of vitiligo seen in patients treated with CIs is thought to be a cross-reaction between the shared antigens in melanoma and normal melanocytes, such as MART1, GP100, or tyrosinase (1). Usually, vitiligo presents progressively, bilaterally, and symmetrically after a few months of immunotherapy and persists beyond the duration of treatment. The development of vitiligo has been proposed to be associated with favorable survival and prognosis (1). Expression of some HLA genes, such as DRB and DQB, is reported to contribute to 30% of vitiligo patients (19). Patient H with vitiligo irAE has predisposing HLA DRB1 07:01, DQB1 02:02, and DQB1 03:19, the transcription of which to cytoplasmic mRNA has been shown to increase the expression of HLA-DQ protein on the surface of antigen-presenting cells known to promote the autoreactive T cell activation with a known role in vitiligo pathophysiology (18). In otherwise healthy individuals, the onset of vitiligo is presumably due to an environmental insult and in our patient, we suggest that immunotherapy is the possible culprit. Moreover, our patient has additional vitiligo-linked HLAs, including DRB1 07:01 and A 02:179, according to the reported meta-analysis (20).

The overall incidence of hepatitis induced by immunotherapies has been reportedly low ~2–15%, and CTLA4 inhibitors have been reported to be associated with more cases of immune-mediated hepatitis (IH) than PD1/PDL1 inhibitors (49). Generally, IH leads to a hepatocellular injury with abnormal findings in liver function test (LFT). Patient I with hepatitis had HLA DRB1^*^04:01 with a well-reported association with IH, specifically in the Caucasian population, in the literature (22, 23).

Understanding HLA inherence in immunotherapy-associated irAEs is an important first step in the risk stratification of patients who may indeed benefit from HLA-specific modulating immune response treatments to provide the full benefit of a completed course of treatment in the relevant patient population. Although such intervention may be on the horizon and is promising, the current level of understanding warrants further research beyond the presented pilot study to further explore the role of HLA-specific TCR targeting molecules to modulate immune response. The HLA-specific TCR targeting molecules have entered clinical use for the treatments of solid malignancies, including uveal melanoma. The consideration to extend the application of such treatment strategies to the irAEs in patients with HLA-specific solid tumors would warrant risk stratification and biomarker selection. An example is tebentafusp, which is an approved treatment for uveal melanoma by targeting HLA A^*^02:01 to trigger T cell immune response. In the future, it may be possible to consider a similar approach in patients with HLA-specific solid tumors presenting with CI-associated irAE to treat the underlying disease while ameliorating or evading irAE. However, targeting HLA-specific TCR molecules has only been used clinically in solid tumors and chronic viral infections thus far (50–52).

Although the presented work is limited by the number of patients, it is to be mentioned that all the patients had DQB1 03. This enriched presence of the DQB1 03 allele, in light of its strong link with autoimmune diseases including BP, can present an opportunity for follow-up studies with a larger number of patients to further investigate HLA as a biomarker to stratify the risk of irAEs in patients on immunotherapy. Additionally, the co-inherence of HLA DRB3 and DPB1, which data show to be frequently found in patients with autoimmune conditions, is seen in seven of nine presented patients. It is noted that the limited number of patients in the presented work would not allow such interpretation of our patients. However, the seven aforementioned patients also exhibited an otherwise rare HLA allele; HLA DRB, which is reported in 17% of the general population. Finally, all of our nine patients were found to share the same HLA haplotype region; HLA DRB1, DQA1, and DQB, which encode proteins that are found to play a key role in presenting antigens to CD4+ T cells in the autoimmune disease processes (53). Once again, the pattern of an enriched presence of HLA allele or haplotype in our patients, although limited in number, can be observed as a potential role that HLA typing can play in irAR risk stratification in patients in whom immunotherapy is lifesaving. That being said, further studies with a larger number of patients are warranted to investigate the significance and analyze the benefit of HLA typing as a risk-stratifying tool at the point of care (Table 1).

The authors acknowledge that the presented work is one step along the collective endeavor toward what precision medicine may 1 day provide.

## Conclusion

These nine patients highlight a spectrum of irAEs with various severities in oncology patients on immunotherapy, for whom such treatment was considered lifesaving. Therefore, risk stratifying these patients to continue immunotherapy in the face of such irAEs became a challenge in clinical decision-making at the point of care. While clinical information and related serologic tests were used in this process, we also applied the HLA typing to assess risk. In the process, we learned that our patients had (a) more than one HLA allele linked to their related irAEs and (b) an enriched presence of certain HLA alleles or haplotype regions competed with the general population. Although we present a limited number of patients here, the data suggest in favor of HLA typing as a risk-stratifying tool in addition to the clinical information and related serologic test to assess irAEs of immunotherapy in oncology patients, for whom this treatment is considered to be lifesaving. Currently, there is no standard method to risk stratify patients for potentially fatal toxicities or early detection of irAEs. The authors acknowledge that follow-up studies with larger data are warranted to fulfill the criteria; however, the presented data are a step toward what precision medicine may 1 day offer.

## Data availability statement

The original contributions presented in the study are included in the article/supplementary material, further inquiries can be directed to the corresponding author.

## Ethics statement

Ethical approval was not required for the study involving humans in accordance with the local legislation and institutional requirements. Written informed consent to participate in this study was not required from the participants or the participants' legal guardians/next of kin in accordance with the national legislation and the institutional requirements. Written informed consent was obtained from the individual(s) for the publication of any potentially identifiable images or data included in this article.

## Author contributions

BD: Conceptualization, Data curation, Formal analysis, Investigation, Methodology, Project administration, Writing – original draft, Writing – review & editing. SG: Writing – original draft, Writing – review & editing. EN: Writing – original draft, Writing – review & editing. DT: Formal analysis, Investigation, Writing – review & editing. RS: Formal Analysis, Investigation, Writing – review & editing. LD: Formal analysis, Supervision, Writing – review & editing.
